# Cerebral microhaemorrhage in COVID-19: a critical illness related phenomenon?

**DOI:** 10.1136/svn-2020-000652

**Published:** 2020-11-18

**Authors:** Luke Dixon, Cillian McNamara, Pritika Gaur, Dermot Mallon, Christopher Coughlan, Francesca Tona, Wajanat Jan, Mark Wilson, Brynmor Jones

**Affiliations:** 1 Department of Neuroradiology, Imperial College Healthcare NHS Trust, London, UK; 2 Department of Cardiac Intensive Care, Imperial College Healthcare NHS Trust, London, UK; 3 Department of Neurosurgery, Imperial College Healthcare NHS Trust, London, UK

**Keywords:** blood flow, brain, haemorrhage, infection, inflammation

## Abstract

**Background:**

Cerebral microhaemorrhages are increasingly being recognised as a complication of COVID-19. This observational retrospective study aims to further investigate the potential pathophysiology through assessing the pattern of microhaemorrhage and clinical characteristics of patients with COVID-19 and microhaemorrhage. By comparing with similar patterns of microhaemorrhage in other non-COVID-19 disease, this study aims to propose possible common pathogenic mechanisms.

**Methods:**

A retrospective observational case series was performed identifying all patients with COVID-19 complicated by cerebral microhaemorrhage on MRI. The distribution and number of microhaemorrhages were recorded using the microbleed anatomical scale, and patients’ baseline characteristics and salient test results were also recorded.

**Results:**

Cerebral microhaemorrhages were noted to have a predilection for the corpus callosum, the juxtacortical white matter and brainstem. All patients had a preceding period of critical illness with respiratory failure and severe hypoxia necessitating intubation and mechanical ventilation.

**Discussion:**

This study demonstrates a pattern of cerebral microhaemorrhage that is similar to the pattern reported in patients with non-COVID-19 related critical illness and other causes of severe hypoxia. This raises questions regarding whether microhaemorrhage occurs from endothelial dysfunction due the direct effect of SARS-CoV-2 infection or from the secondary effects of critical illness and hypoxia.

## Introduction

Numerous reports have highlighted different neurological complications of COVID-19, including cerebrovascular disease, encephalopathy and peripheral neuropathy.^1–4^ The pathogenesis of these varied presentations is unknown. It remains to be seen whether neurological disease in COVID-19 is directly caused by SARS-CoV-2 infection, indirect as a sequala of critical illness or entirely coincidental.^5 6^

White matter microhaemorrhage (MH) has been reported as a radiological presentation in COVID-19.^4^ Cerebral MHs detected on susceptibility-weighted MRI are a radiological–pathological correlate of tiny focal bleeds in the brain that occur due to transient focal disruptions in the blood–brain barrier.^7^ Many different disease processes can lead to MH often with distinctive distributions.^8^ Here we report the clinical characteristics and radiological pattern of MH in a series of patients with severe COVID-19 and draw comparisons with previous studies to cast light on potential pathophysiological mechanisms.

## Methods

This retrospective observational case series was approved by local ethics review. Patient data were anonymised at the point of analysis, and the need for informed consent was waived. Patients who underwent brain MRI between 1 April 2020 and 1 June 2020 with a confirmed diagnosis of SARS-CoV-2 infection (on reverse transcriptase PCR assay of an airway tract sample) were identified using searches of electronic health records at a university-affiliated hospital. MRI examinations were performed according to a routine brain protocol, which included a susceptibility-weighted sequence. The susceptibility-weighted sequence technique varied across scanners. Two radiologists collectively reviewed the imaging, and a third radiologist reviewed the cases independently. If there was disagreement, cases were reviewed jointly for consensus opinion. Patients were included in the case series if they had tested positive for SARS-CoV-2 prior to the MRI and had MH on their MRI, which was separate to other CNS pathology such as ischaemic stroke. The location and volume of MH was scored according to the microbleed anatomical scale (modified to also include the middle cerebellar peduncles) and manually mapped to a simplified brain diagram. These diagrams were used to generate a heatmap of composite MH distribution across all patients, as described elsewhere.^9^ The MH heatmap was generated using Heatmapper, an R-based online tool.^10^ The presence of T2-weighted signal abnormality and restricted diffusion was also recorded. We reviewed the electronic health records of all patients included in the case series and extracted salient laboratory results and investigations.

## Results

We identified 30 MR brain scans performed on 28 adult patients with confirmed COVID-19 between 1 April and 1 June 2020. Of these, 10 patients were noted to have MH distinct from other central nervous system (CNS) pathology.

### Radiology

**Figure 1:**
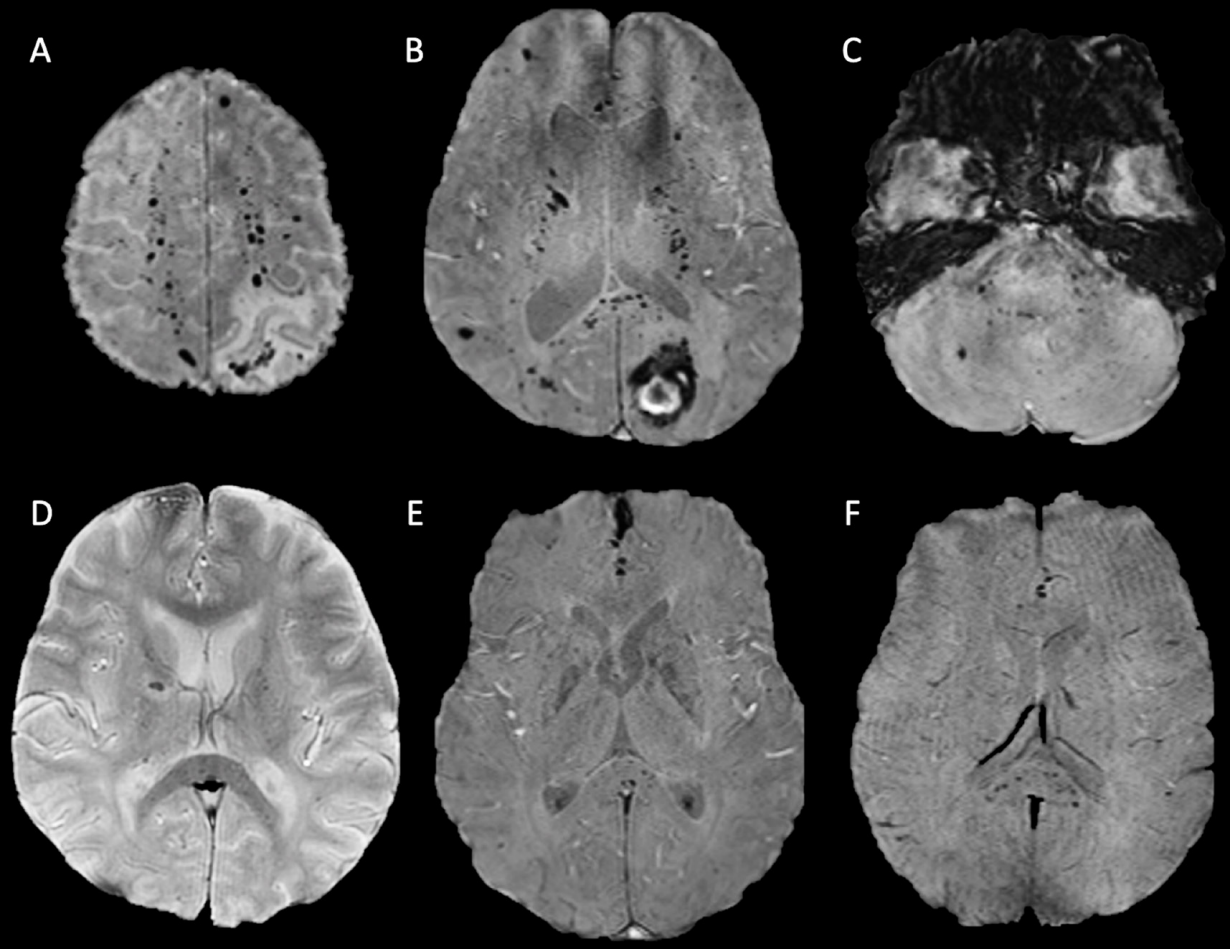
Axial susceptibility-weighted MRI sequences of patient 4 (A–C) and patients 2 (D), 5 (E) and 10 (F). All demonstrating microhaemorrhages in the splenium of the corpus callosum and juxtacortical and subcortical white matter. Patients 4 (A–C) and 2 (D) also both exhibit microhaemorrhages in the internal capsule. The axial image of the posterior fossa in patient 4 (C) demonstrates further microhaemorrhages in the pons, middle cerebellar peduncles and cerebellum.

**Figure 2:**
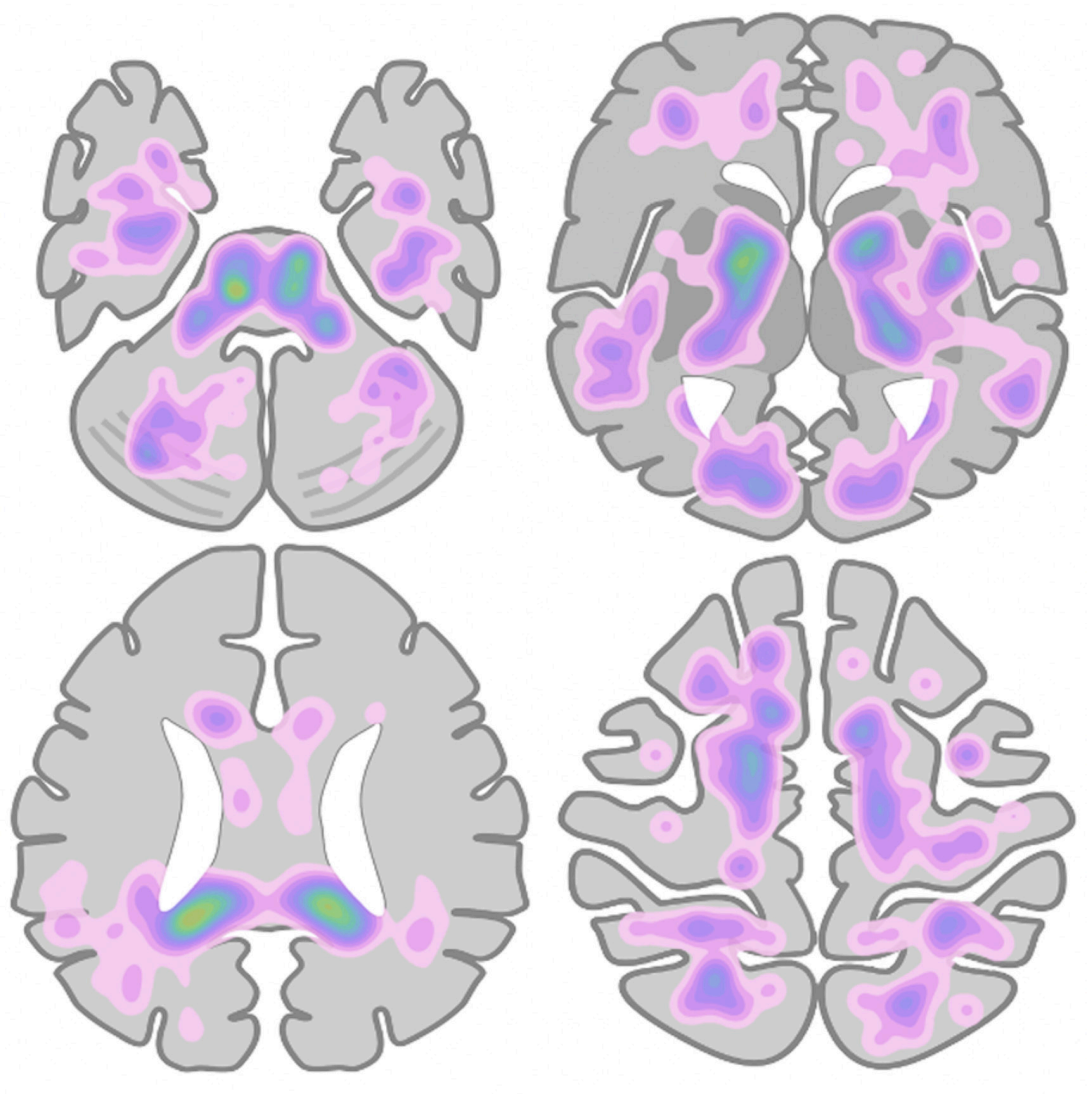
Heatmap of microhaemorrhage density and distribution across all patients measured, based on manual labelling of microhaemorrhage locations on a simplified brain schematic.

MRI was of diagnostic quality in 9 of the 10 patients. In one patient, imaging was degraded by motion artefact, and therefore, MH could not be quantified accurately. All 10 patients demonstrated MH in the corpus callosum with a predilection for the splenium. In the nine MRIs included in the quantitative assessment (table 1), the median number of MH was 35 (range 16–270). In addition to the corpus callosum, MHs were predominantly in the juxtacortical and subcortical white matter of both cerebral hemispheres, particularly the parietal lobes, and in the cerebellum and brainstem. Figure 1 demonstrates example images, and figure 2 shows a heatmap of MH distribution across all nine scored patients. We observed abnormal areas of high T2-weighted signal in the supratentorial white matter in two scans, and one MRI also showed separate macroscopic haemorrhages in the occipital and parietal lobes. No MRI demonstrated restricted diffusion. No repeat follow-up imaging had been performed at the time of publication.

**Table 1:** Microhaemorrhage score and additional imaging findings for each patient (1–10)

	1	2	3	4	5	6	7	8	9	10	Median
Brainstem	5	5	5	7	0	1	–	0	4	0	**4.00**
Cerebellum	1	2	2	19	4	3	–	0	0	2	2.00
Middle cerebellar peduncle	0	4	3	7	0	0	–	0	0	0	0.00
Basal ganglia	0	2	3	5	2	0	–	0	1	1	1.00
Thalamus	0	0	0	1	0	0	–	0	0	0	0.00
Internal capsule	1	2	0	19	2	2	–	0	6	0	2.00
External capsule	0	9	0	7	0	0	–	0	2	0	0.00
Corpus callosum	17	2	12	52	5	4	–	1	15	14	**12.00**
Genu	5	16	1	7	1	0	–	0	7	2	2.00
Body	0	2	1	8	0	1	–	1	0	0	1.00
Splenium	12	4	10	37	4	3	–	0	8	12	**8.00**
Deep periventricular white matter	0	10	0	20	0	5	–	2	0	0	0.00
Frontal	0	0	0	51	0	16	–	2	4	3	2.00
Parietal	0	18	0	35	4	7	–	2	6	9	**6.00**
Temporal	0	8	0	30	3	2	–	9	1	3	3.00
Occipital	0	4	0	16	1	4	–	0	0	3	1.00
Insula	0	4	0	1	0	0	–	0	0	0	0.00
T2 signal change	No	Yes	No	Yes	No	No	No	No	No	No	
Restricted diffusion	No	No	No	No	No	No	No	No	No	No	
Macroscopic haemorrhage	No	No	No	Yes	No	No	No	No	No	No	
Total	24	70	25	270	21	44	–	16	39	35	35.00

Bold values refer to theareas with the greatest number of microhaemorrhages.

### Clinical

Patient clinical features are summarised in table 2. Median age was 56 years (range, 48–66 years), and eight patients were male. All patients required critical care admission for intubation and mechanical ventilation due to type 1 respiratory failure (T1RF) from acute respiratory distress syndrome (ARDS), and all patients had recorded episodes of severe hypoxia and hypercapnia.

**Table 2:** Summary of patient demographics and clinical features

	1*	2	3	4	5	6	7	8	9	10	Median
Age (years)	52	51	60	62	55	57	66	54	66	48	56.00
Sex	Female	Male	Male	Male	Male	Male	Male	Female	Male	Male	–
Premorbid condition											
Hypertension	Yes	Yes	Yes	–	–	Yes	Yes	–	–	–	–
Chronic kidney disease	Yes	–	Yes	–	–	–	Yes	–	–	–	–
Diabetes mellitus	–	–	–	–	–	Type 2	Type 2	Type 1	–	Type 2	–
Obesity	–	–	–	–	Yes	–	Yes	–	–	–	–
Hypercholesterolaemia	–	–	–	–	–	Yes	Yes	–	–	Yes	–
Respiratory condition	–	–	–	–	–	–	COPD	–	–	–	–
Other	–	–	Multiple myeloma	Situs inversus		–	IHD	–	Polymyalgia	–	–
ARDS	Yes	Yes	Yes	Yes	Yes	Yes	Yes	Yes	Yes	Yes	–
Reason for ITU admission	T1RF	T1RF	T1RF	T1RF	T1RF	T1RF	T1RF	T1RF	T1RF	T1RF	–
Length of intubation (days)	12	29	25	25	22	21	43	33	38	5	25.00
ECMO duration (days)	–	–	–	–	–	–	–	–	–	14	0
Haemodialysis	CVVHDF	–	CVVHDF	–	CVVHDF	CVVHDF	CVVHDF	CVVHDF	CVVHDF	–	–
Heparinisation	Yes	Yes	Yes	Yes	Yes	Yes	Yes	Yes	Yes	Yes	–
Neurological presentation	AMS	AMS	AMS	Right-side weakness	AMS	AMS	AMS	Abnormal ventilation	Tremors	Seizures	–
Highest recorded blood pressure	190/70	150/112	110/80	–	143/96	208/69	163/74	172/81	137/77	142/91	–
Biochemistry and haematology, worst value											
pH (7.35–7.45)	–	**7.12**	**7.15**	**7.05**	**7.05**	**7.09**	**7.19**	**6.95**	**7.13**	**7.15**	7.12
PaCO_2_ (4.7–6 kPa)	–	**11.3**	**9.9**	**19.4**	**11.4**	**14.2**	**7.9**	**17.7**	**14.9**	**16.1**	14.20
PaO_2_ (10.0–13.3 kPa)	–	**5.9**	**8.4**	**6.6**	**4.1**	**5.3**	**6.5**	**8.1**	**8.2**	**8.1**	6.60
LDH (125–243 units/L)	**609**	**345**	**774**	**410**	**1096**	**663**	**533**	229	**591**	**837**	600.00
Hb (115–155 g/L)	**72**	**68**	**68**	**71**	**68**	**73**	**63**	**64**	**66**	**91**	68.00
WCC (4.2–10.6×10^9^/L)	**14.4**	**19.9**	**31.6**	**20.4**	**29.9**	**14.6**	**19.3**	**25.6**	**24.3**	**31.3**	22.35
Lymphocytes (1.1–3.6×10^9^/L)	2.2	**0.4**	**0.6**	**0.3**	**0.6**	**0.5**	**0.2**	**0.3**	**0.3**	**0.7**	0.45
Neutrophils (2.0–7.1×10^9^/L)	**10.3**	**17.3**	**15.9**	**18.4**	**15.7**	**16.3**	**17.8**	**24.1**	**22.1**	**25.6**	17.55
Platelets (highest/lowest) (130–370×10^9^/L)	**537/438**	**732/**189	318/**100**	285/142	**525**/175	342/143	298/**102**	**547**/138	**733**/369	**871**/280	537/143
Urea (2.5–7.8)	**44.3**	**14.9**	**51**	**20.3**	**41.3**	**49.8**	**42.6**	**26.9**	**43.3**	7.7	41.95
CRP (<5 mg/L)	**49**	**426**	**342**	**349**	**401**	**365**	**220**	**284**	**266**	**175**	313.00
Ferritin (20–300 ng/mL)	**2714**	**3607**	**3762**	**7569**	**4003**	**739**	**710**	**526**	**4916**	12 592	3684.50
PT (12.8–17.4 s)	15.6	**18.5**	**20**	**17.5**	14.9	15.5	**19.8**	**18.2**	16.2	15.3	16.85
Fibrinogen (1.9–4.3 g/L)	**5.1**	**8.6**	**9.6**	**8.7**	4.3	**10.3**	**7.5**	**8.6**	**7.9**	**7.5**	8.25
D-dimer (<500 ng/mL)	2010	20 000	20 000	7569	6309	20 000	12 064	5569	13 148	7060	9816.50
Haematocrit (0.39%–0.50%)	0.224	0.424	0.44	0.3	0.41	0.4	0.36	0.34	**0.289**	0.41	0.38
DIC score	3	4	4	3	4	4	4	4	4	4	4.00
Systemic thrombosis	No	No	No	No	No	No	IJV thrombus	No	DVT	No	
Extent of consolidation on thoracic imaging	–	Severe	Severe	Severe	Severe	Severe	Severe	Severe	Severe	Severe	
MRI head day from admission	24	38	37	32	29	24	53	59	58	41	37.50
MARS	24	70	25	270	21	44	–	16	39	35	60.44

Bold values highlight the areas with the greatest median number of microhaemorrhages.

*Patient 1 has incomplete biochemistry information due to their initial care being at an external institution.

AMS, altered mental state; ARDS, acute respiratory distress syndrome; COPD, chronic obstructive pulmonary disease; CRP, c-reactive protein; CVVHDF, continuous venovenous haemodiafiltration; DIC, disseminated intravascular coagulation; ECMO, extracorporeal membrane oxygenation; Hb, haemoglobin; IHD, ischaemic heart disease; IJV, internal jugular vein; ITU, intensive therapy unit; LDH, lactate dehydrogenase; MARS, microbleed anatomical scale; Pa02, partial pressure of oxygen; PaC02, partial pressure of carbon dioxide; PT, prothrombin time; T1RF, type 1 respiratory failure; WCC, white cell count.

The median time to MRI scan was 37.5 days after admission (range: 24–59 days). Indications included altered mental state (6/10), weakness (1/10), seizure (1/10), tremors (1/10) and an abnormal brainstem pattern of ventilation (1/10). Seven out of 10 patients required haemodialysis or haemofiltration for acute kidney injury. One patient needed extracorporeal membrane oxygenation (ECMO) for refractory T1RF. All patients received antithrombotic therapy with heparinisation.

### Biochemistry and haematology

All patients had recorded periods of acidosis, raised lactate dehydrogenase, anaemia, lymphopaenia and neutrophilia. Nine of the 10 patients had episodes of acute kidney injury. All patients had raised CRP, ferritin and high D-dimer. Nine patients had high fibrinogen, and five patients had mildly prolonged prothrombin time.

### Clinical outcome

All patients were alive at the time of this report, and all had been discharged from critical care. At the time of discharge abnormal laboratory findings had improved or normalised for all patients.

### Summary of findings

We present a case series of 10 patients with COVID-19, abnormal neurology and a common distribution of white matter MHs, with a predilection for the splenium of the corpus callosum. This pattern of MH is unusual and distinct from the typical MH distributions in other causes, in particular it would not fit diagnostic criteria for either hypertensive arteriopathy or amyloid angiopathy.^7 8^ This is the first series to specifically look at MH in COVID-19, and it highlights several clinical features. First, the majority of patients were male (80%) and relatively young with a median age of 58 years (range 48–66 years). This is younger than the average age that MH is typically seen in the normal ageing population, 70–76 years.^11 12^ Second, half of the patients had at least one comorbidity including hypertension, chronic kidney disease and diabetes. This observation is expected; it is now well established that certain comorbidities are associated with a greater risk of respiratory failure, critical care admission and death.^13^ The presence of comorbidity was not however a decisive factor for MH as several patients had no significant prior medical history. Finally, the median interval from admission due to COVID-19 and neurological presentation and diagnosis of MH was 37 days. In all cases, there was preceding systemic inflammation and critical illness characterised by ARDS and respiratory failure that required intubation and mechanical ventilation. In all patients, there was also anaemia and in the majority acute kidney injury (90%). Our findings complement two recent case series that found the same MH pattern in a collective total of 16 patients with severe COVID-19. The patients described in these studies also required critical care due to ARDS and severe hypoxia and showed evidence of systemic inflammation, coagulopathy, anaemia and a degree of acute kidney injury.^4 14^ This final observation raises the question whether MH occurs as a direct consequence of SARS-CoV-2 infection or as an epiphenomenon of severe systemic illness.

## Discussion

Cerebral MH occurs secondary to endothelial dysfunction causing focal extravasation of red blood cells into the brain.^7 9^ In COVID-19, there are several different, potentially synergistic mechanisms that may lead to this cerebral endothelial dysfunction. Multisystem endothelialopathy has emerged as a key feature of severe COVID-19, with microthromboses and endothelial inflammation noted in the vessels of the lung, heart, kidneys, liver and small intestine.^15 16^ The cause of this endothelial inflammation in COVID-19 remains unclear, with both direct viral infection and indirect inflammatory-mediated processes proposed.^17 18^ Direct binding of SARS-CoV-2 to the endothelium via the putative ACE-2 receptor has been postulated as one cause. This is supported by a report that demonstrated possible viral elements in the endothelial cells of multiple organs and the observation that ACE-2 receptors are expressed in the brain in both the cerebral endothelium and non-vascular tissue including neuronal cell bodies.^16 19 20^ Whether SARS-CoV-2 directly invades vascular endothelium is however contentious and remains uncertain.^21^ Another proposed effect of ACE-2 receptor downregulation is disruption of the renin-angiotensin system causing cerebrovascular dysautoregulation, altered cerebral perfusion and secondary endothelial dysfunction.^22–24^ The finding of altered cerebral perfusion in critically ill patients with COVID-19 adds weight to this theory, with frontotemporal hypoperfusion noted in a series of 11 patients on MR perfusion imaging.^25^ Interestingly, in a separate isolated case report parieto-occipital hyperperfusion was noted in a patient with COVID-19 and a similar pattern of callosal predominant MH.^26^ This overlaps with recent reports of COVID-19 related posterior reversible encephalopathy syndrome (PRES).^27^ These early suggestions of posterior circulation hyperperfusion perhaps accounts for the splenial and posterior fossa distribution of MH in our series, although MH was not exclusively distributed in the typical areas that PRES involves. Furthermore, although cerebrovascular dysautoregulation appears to be an important factor, there are several indirect causes of endothelialopathy and altered cerebral perfusion that need to be explored beyond direct effects of the virus. The role of systemic inflammation and hypoxia specifically warrant discussion.

Severe COVID-19 is associated with a hyperinflammatory syndrome and cytokine storm, which itself is thought to mediate endothelial dysfunction and coagulopathy.^28^ Circumstantial evidence for this in COVID-19 related MH is the common finding of systemic inflammation and coagulopathy in both ours and other cohorts. One possible explanation for the corpus callosal MH predilection is that the corpus callosum, particularly the splenium, has a relatively high concentration of cytokine and glutamate receptors that may confer greater sensitivity to these inflammatory factors.^29^ Interestingly, the same splenial predominant pattern of MH was reported in a case series of 10 non-COVID-19 critically ill patients all with systemic inflammation, coagulopathy and high D-dimer. The authors suggested an association of endothelial dysfunction, DIC and thrombotic microangiopathy as a common potential cause of MH in critically ill patients.^30^ In our study and across all similar COVID-19 case series published to date, there was evidence of coagulopathy, but no patients met international consensus criteria for overt DIC.^4 14 31^ This aligns with a recent study that suggests that COVID-19 coagulopathy is distinct from DIC.^32^ Of note, no patients in our series had macroscopic evidence of intracranial arterial or venous thrombosis, and only two patients had systemic evidence of venous thrombotic disease.

Comparison with other accepted causes of MH may also help illuminate pathogenesis. A similar distribution of MH has been described in mountaineers who develop high-altitude cerebral oedema (HACE) and in patients with non-COVID-19 related respiratory failure and critical illness.^33–36^ This shared pattern across COVID-related MH and these other disparate groups may suggest an underlying common pathogenesis. Exposure to severe hypoxia is universal in these otherwise distinct cohorts. The mean worst recorded hypoxaemia in our series was a partial pressure of oxygen (PaO_2_) of 6.8, which is equivalent to the PaO_2_ at an altitude of 5000 feet.^37^ The pathophysiology of MH in hypoxia is not fully understood. HACE is thought to result from rapid ascent to high altitudes resulting in acute exposure to hypobaric hypoxia, which leads to disruption of the blood brain barrier, vasogenic oedema and leakage of blood products.^38^ Cerebral capillary hypertension is a postulated cause for this and is thought to occur secondary to disturbances of cerebrovascular autoregulation and/or from impaired cerebral venous return, particularly in the context of hypoxia-induced cerebral vasodilatation.^34 38 39^ The suggested role of venous hypertension in HACE may also be relevant in COVID-19 and other critical illness related MH. In these groups, there are potentially further causes of raised central venous pressure, including positive pressure ventilation, ECMO therapy and haemodialysis.^35^ The impact of these factors is uncertain; however, not all our patients (20%) required multiorgan support and our patient with the greatest burden of MH did not receive either haemodialysis or ECMO. Interestingly, biochemical abnormalities leading to increased vascular permeability are also thought to contribute to blood–brain barrier breakdown in patients with HACE and severe hypoxia. This includes hypoxia-induced release of vascular endothelial growth factor, reactive oxygen species and cytokines.^37 40^

Aside from pathogenesis, the long-term influence of COVID-19 related MH on cognitive, neurological and psychological outcomes are also unknown. In other diseases, MH has been independently associated with cognitive impairment and disability.^41^ In our series formal cognitive assessment had not yet been undertaken but several of the patients still reported ongoing issues with memory at discharge.

This study adds weight to previous work demonstrating a common but unusual pattern of cerebral MH in critically ill patients with COVID-19. As a small case series, there are however limitations. Patients were retrospectively selected for the specific finding of MH creating a selection bias. Second, as imaging was performed as part of routine clinical care, MRI scans were performed at different time points within the patients clinical course. Resultantly, MRIs were likely performed several days to weeks after the neurological event as the neurological presentations were probably concealed by sedation and only apparent on weaning. Finally, there were differences in the susceptibility-weighted sequence employed that had different sensitivities to MH limiting comparison between patients.

## Conclusion

In conclusion, we present further evidence of a distinctive pattern of MH in COVID-19. Whether direct effects of SARS-CoV-2, systemic inflammation, coagulopathy or hypoxia mediate the MHs observed in COVID-19 remains unclear. The observation that the same pattern of MH is also seen in high altitude exposure and different types of critical illness perhaps implies that COVID-19 associated MH is a more phenomenon of hypoxia and critical illness as opposed to a unique feature of SARS-CoV-2 infection. Various discussed mechanisms may contribute to a final common pathway of endothelial dysfunction and blood–brain barrier breakdown. Further larger series and prospective case–control studies are needed to better understand the pathogenesis of COVID-19 related MH. Long-term follow-up is also needed to assess the impact on cognitive and functional outcome.
